# G Protein-coupled Receptor (GPCR) Reconstitution and Labeling for Solution Nuclear Magnetic Resonance (NMR) Studies of the Structural Basis of Transmembrane Signaling

**DOI:** 10.3390/molecules27092658

**Published:** 2022-04-20

**Authors:** Haoyi Ge, Huixia Wang, Benxun Pan, Dandan Feng, Canyong Guo, Lingyun Yang, Dongsheng Liu, Kurt Wüthrich

**Affiliations:** 1 iHuman Institute, ShanghaiTech University, Shanghai 201210, China; gehy@shanghaitech.edu.cn (H.G.); wanghx1@shanghaitech.edu.cn (H.W.); panbx@shanghaitech.edu.cn (B.P.); fengdd@shanghaitech.edu.cn (D.F.); guocy@shanghaitech.edu.cn (C.G.); yangly@shanghaitech.edu.cn (L.Y.); liudsh@shanghaitech.edu.cn (D.L.); 2 School of Life Science and Technology, ShanghaiTech University, Shanghai 201210, China; 3 Department of Integrative Structural and Computational Biology, Scripps Research, La Jolla, CA 92037, USA; 4 Institute of Molecular Biology and Biophysics, ETH Zürich, Otto-Stern-Weg 5, 8093 Zürich, Switzerland

**Keywords:** G protein-coupled receptors, ^19^F-NMR, membrane mimetics, stable-isotope labeling, in-membrane chemical modification, amino-acid-specific NMR labeling, sequence-specific NMR labeling

## Abstract

G protein-coupled receptors (GPCRs) are a large membrane protein family found in higher organisms, including the human body. GPCRs mediate cellular responses to diverse extracellular stimuli and thus control key physiological functions, which makes them important targets for drug design. Signaling by GPCRs is related to the structure and dynamics of these proteins, which are modulated by extrinsic ligands as well as by intracellular binding partners such as G proteins and arrestins. Here, we review some basics of using nuclear magnetic resonance (NMR) spectroscopy in solution for the characterization of GPCR conformations and intermolecular interactions that relate to transmembrane signaling.

## 1. Introduction

G protein-coupled receptors (GPCRs) are seven-transmembrane integral membrane proteins, and they are the largest family of druggable proteins in the human genome [1]. More than 30% of all prescription drugs approved by the U.S. Food and Drug Administration (FDA) target GPCRs [2], and these drugs are utilized in a wide range of therapeutic areas, including cardiovascular, metabolic, neurodegenerative, psychiatric, and infectious diseases and cancer [3].

**Figure 1:**
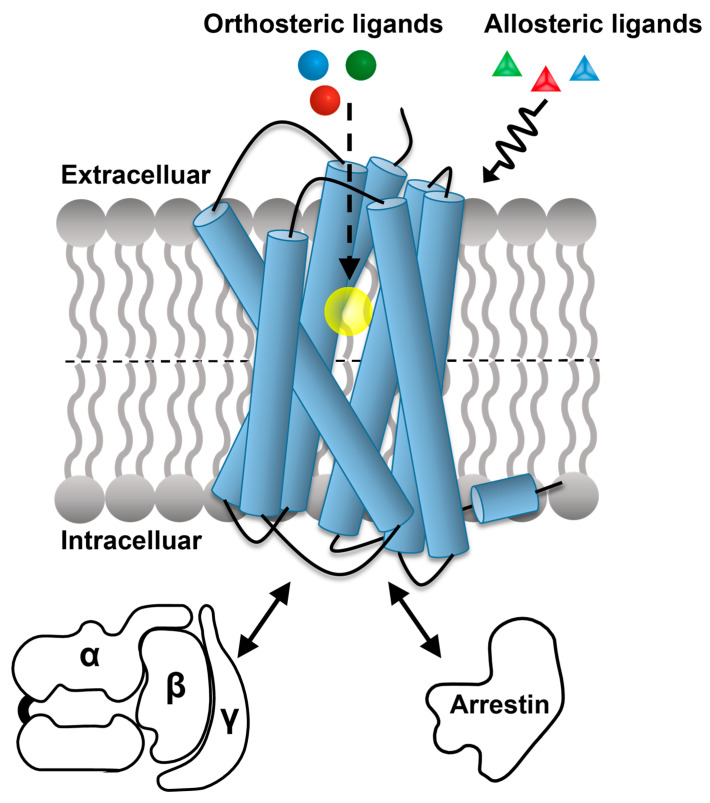
Overview of the intermolecular interactions involved in cellular signaling by GPCRs. The orthosteric binding pocket (indicated as a yellow ball) is usually targeted by endogenous ligands (indicated as small colored balls), which interact with the orthosteric binding pocket; they alter the activation state of the receptors according to their efficacy as full agonists, partial agonists, antagonists or inverse agonists. Allosteric ligands (indicated as triangles) typically bind to sites that are spatially distinct from the orthosteric binding pocket and modulate the affinity and/or efficacy of the ligand bound to the orthosteric site. Representative intracellular interaction partners of GPCRs are heterotrimeric G proteins (α, β, γ) and arrestins. Biased GPCR ligands preferentially activate either the G protein or the arrestin signaling pathways. (Adapted from Figure 2 in reference [12]).

GPCRs mediate most of the cellular responses to external stimuli, including neurotransmitters, hormones and photons [4]. An “orthosteric” binding site is the primary target for endogenous substances and a wide range of extrinsic ligands, which alter the activation state of the receptors according to their efficacy as full agonists, partial agonists, antagonists or inverse agonists [5]. Allosteric ligands typically bind to sites that are spatially distinct from the orthosteric binding pocket and modulate the binding affinity and/or efficacy of the ligand bound to the orthosteric site [6]. Biased GPCR ligands preferentially activate one of the signaling pathways (Figure 1) [7,8]. The intracellular interaction partners of GPCRs include heterotrimeric G proteins and arrestins [9]. Upon G protein activation, the G_α_ subunit dissociates from the G_βγ_ subunits and induces the production of second messengers, such as cAMP [4]. G_βγ_ modulates downstream signaling networks, including ion channels and phospholipases [10]. GPCR signaling through arrestins is initiated by the phosphorylation of the receptor C-terminal tail by a G protein-linked kinase; thus, recruited arrestins block G protein-mediated signaling and direct the receptors to internalization [11].

Although most often targeted directly by small molecule drugs [13,14], GPCRs have also become interesting targets for antibodies and antibody fragments [15,16]. Antibodies can bind tightly and specifically to their targets, even in highly complex environments [17], and have thus been used to deliver bioactive compounds to GPCR sites of interest for diagnostic and therapeutic applications [18]. 

This review addresses basic principles of the use of nuclear magnetic resonance (NMR) spectroscopy in solution to investigate interactions with orthosteric and allosteric ligands of different efficacies, and to study the function-related impact of bound ligands on the structure and dynamics of GPCRs. 

## 2. Preparation of GPCRs for NMR Studies

Membrane proteins perform their physiological functions in the phospholipid cell membranes. For NMR experiments in aqueous solutions, GPCRs must be reconstituted in membrane mimics that stabilize the native conformation and enable the preparation of solutions with a sufficiently high concentration for the detection of the NMR signals. There is a long history of the optimization of lipids and detergents for GPCR isolation, purification and in vitro characterization [19]. The solubilizing approach usually impacts ligand binding as well as the GPCR’s structure and dynamics. In this chapter, we survey the major techniques in use for the preparation of GPCR solutions, which has been and continues to be a bottleneck for NMR studies of membrane proteins.

### 2.1. Solubilization of GPCRs in Detergent Micelles

**Figure 2:**
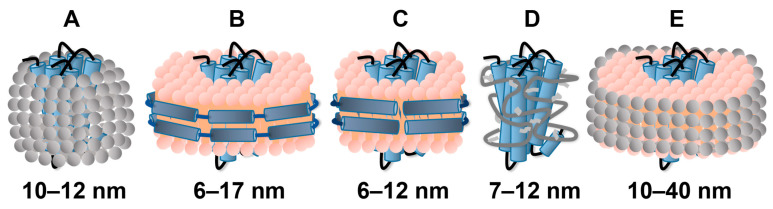
Cartoon representation of GPCRs embedded in five different membrane mimics in use for reconstitution and solubilization for NMR experiments in solution. (**A**) GPCR (blue) stabilized in detergent micelles (gray). (**B**) GPCR (blue) embedded in a nanodisc composed of a lipid bilayer (pink) and membrane scaffold proteins (dark blue). (**C**) GPCR (blue) embedded in a saposin A nanoparticle composed of a lipid bilayer (pink) and saposin A molecules (dark blue). (**D**) GPCR (blue) embedded in amphipathic polymers or amphipols (gray). (**E**) GPCR (blue) embedded in bicelles composed of long-chain lipids (pink) and short-chain lipids or detergent molecules (gray). (Adapted from Figures 2, 4 and 5 of reference [21]; the size ranges indicated below the individual cartoons are taken from reference [23]).

The amphiphilic character of detergents is evident in their structures, which consist of hydrophobic tails and polar head groups [20]. Above the critical micelle concentration (CMC), self-association occurs and micelles form. Since GPCRs contain both hydrophobic and hydrophilic regions, detergents can stabilize the receptors and prevent aggregation and precipitation in aqueous solutions (Figure 2A) [21]. Detergents are extensively used for extracting and solubilizing membranes in crystallography [22]; for NMR in solution, they are also extensively used because of the relatively small size and near-spherical shape of the GPCR-containing micelles (Figure 2). 

Comparison of β_2_AR in *n*-dodecyl β-d-maltoside (DDM) and 2,2-didecylpropane-1,3-bis-β-d-maltopyranoside (LMNG) micelles indicated that higher off-rates in DDM tend to facilitate ligand-induced conformational exchanges between different functional states [24]. Molecular dynamics simulations showed that LMNG is more tightly packed to the receptor, which would stabilize the given receptor conformations and reduce the dynamics of the receptor–detergent complexes [25].

Overall, in vitro studies of the GPCR structure and function are widely pursued with reconstitution in detergent micelles, which represent a highly disordered environment when compared to the native membrane. High detergent concentrations may also interfere with ligand and G protein binding [21]. Major efforts are therefore geared at complementing experiments with detergent micelles by using alternative solubilization methods (Figure 2) for exploring the GPCR structure and function [19,20,21,22,24,25].

### 2.2. Reconstitution of GPCRs in Nanodiscs

Nanodiscs (Figure 2B) are nano-sized, self-assembled, disc-shaped lipid bilayer structures [19]. The most widely used nanodiscs are composed of phospholipids and encircling amphipathic helical proteins, termed membrane scaffold proteins (MSP) [26]. Nanodiscs with membrane proteins can be assembled from a detergent-solubilized mixture of all components by the removal of the detergent via adsorption on hydrophobic bio-beads.

The major advantages of the nanodiscs are the absence of detergent molecules and the ability to maintain their integrity and shape upon dilution [19]. The state of membrane proteins in nanodiscs is expected to be close to that in the native membrane. Staus et al. [27] showed that, compared with detergent-reconstituted β_2_AR, β_2_AR in nanodiscs had greatly enhanced levels of basal (constitutive) activity and displayed increased sensitivity to agonist activation. Mizumura et al. [28] found that the docosahexaenoic acid chains in nanodiscs redistribute the conformations of the equilibria of the A_2A_ adenosine receptor (A_2A_AR) toward those preferable for G protein binding, and that the population shift of the equilibrium causes enhanced G protein activation by A_2A_AR.

Saposin A nanoparticles are disc-like complexes formed by the sphingolipid-activating protein and lipids (Figure 2C) [29]. Saposin A is more rigid and forms much smaller lipid particles than MSP nanodiscs; the size of saposin nanodiscs can also be influenced by variation in the ratio of saposin A and lipids [30,31]. Studies of the turkey β_1_AR receptor in saposin A nanoparticles suggested that the receptor retains the ability to functionally interact with binding partners, and dynamic exchange between several conformations was also observed [32]. Due to its containing disulfide bonds, the production of saposin A is less straightforward than that of MSP, which has so far limited widespread use of this system [33]. 

### 2.3. Use of Amphipols or Bicelles for GPCR Solubilization

Amphipols are amphipathic polymers that allow membrane protein solubilization and stabilize the native state [34] (Figure 2D). Amphipols have a pronounced hydrophobic character, which tends to enhance interactions of ligands with the surfactant molecules. For example, a study of the leukotriene receptor 2 (BLT2) in amphipols revealed that heptadecanoid 12S–hydroxyheptadeca-5Z,8E,10E-trienoic acid (12-HHT) shows a higher non-specific binding than leukotriene B4 (LTB4) [35]. Amphipol-trapped GPCRs have been reported to essentially maintain their pharmacological properties, so that they can be used to further investigate the molecular mechanisms underlying GPCR signaling processes [36]. Limitations may arise because amphipols are sensitive to environmental change, and their solubility is dependent on the pH and presence of multivalent cations [37]. 

Bicelles are disc-shaped structures commonly comprised of long-chain phospholipids and either detergents or short-chain phospholipids [38] (Figure 2E). An advantage of this system is its size scalability through variation in the molar ratio between the long-chain and short-chain lipid components [33]. Park et al. [39] successfully reconstituted the CXCR1 chemokine receptor into bicelles to immobilize and align the protein for solid-state NMR experiments. However, when using detergent bicelles as the membrane mimetic, the concentration of monomeric detergent in the NMR samples can be substantial; globular proteins, such as GPCR-binding partners or the extramembranous regions of a receptor, may thus be inactivated. It also appears to be difficult to achieve the needed optimal ratio of lipids and detergents for solution NMR [23].

## 3. ^19^F-NMR with Observation of Extrinsic Probes Attached to GPCRs

**Figure 3:**
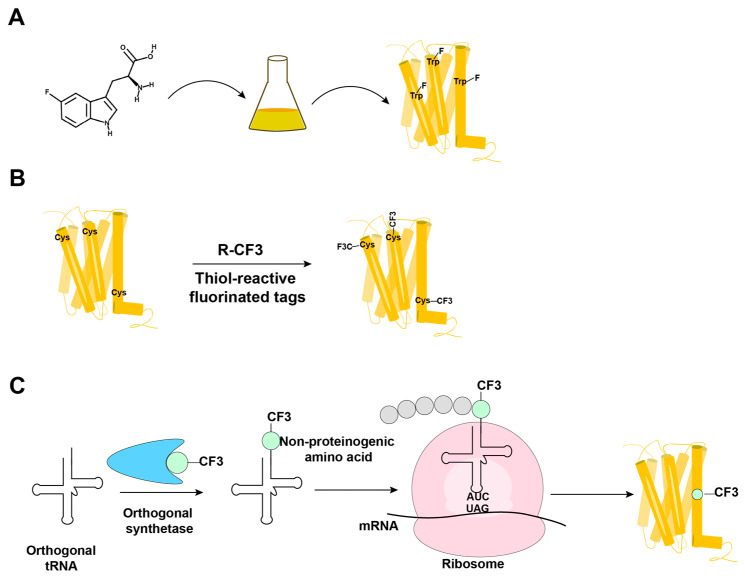
Overview of the methods in use for incorporation of ^19^F-NMR labels into GPCRs. (**A**) Biosynthetic incorporation by adding fluorinated amino acids, such as 5F-Trp, to the expression system; all Trp residues in the protein are then labeled with ^19^F. (**B**) Post-translational chemical modification by reacting the GPCR with thiol-reactive fluorinated tags; all reagent-accessible Cys residues are then labeled with the fluorinated tag. (**C**) Genetic labeling using an extrinsic orthogonal tRNA/aminoacyl-tRNA synthetase pair to incorporate non-proteinogenic ^19^F-labeled amino acids at positions defined by a TAG amber codon.

^19^F-NMR has long been used for studies of complex biological systems, since ^19^F has no natural background signals and displays high sensitivity toward changes in its microenvironment. As fluorine is not a natural component of proteins, it is essential to develop ever-improved methods to incorporate fluorine probes into GPCRs, either during expression or by post-translational chemical modification (Figure 3) [40,41,42,43,44].

In biosynthetic incorporation, all residues of one amino acid type can be replaced by its fluorinated analogue, providing “amino-acid-specific ^19^F-labeling” (Figure 3A). Fluorinated amino acids are fed to the expression host by including a high concentration of the fluorinated amino acid in the growth medium (Figure 3A) [41,45,46]. This approach may be limited by the fact that high concentrations of ^19^F-containing amino acids can inhibit cell growth [47]. Induction of the amino acid auxotrophy in nonauxotrophic bacterial strains by shutting down selected amino-acid-specific biosynthesis pathways with specific inhibitors has also been used for ^19^F-labeling [48,49]. Tryptophan residues are highly present at the hydrophobic interfaces of protein–protein complexes, which makes fluorotryptophan attractive for NMR studies of membrane proteins [50,51]. The use of fluorinated indole as a fluorotryptophan precursor has been described as an inexpensive alternative for obtaining tryptophan-specific labeling [47].

**Figure 4:**
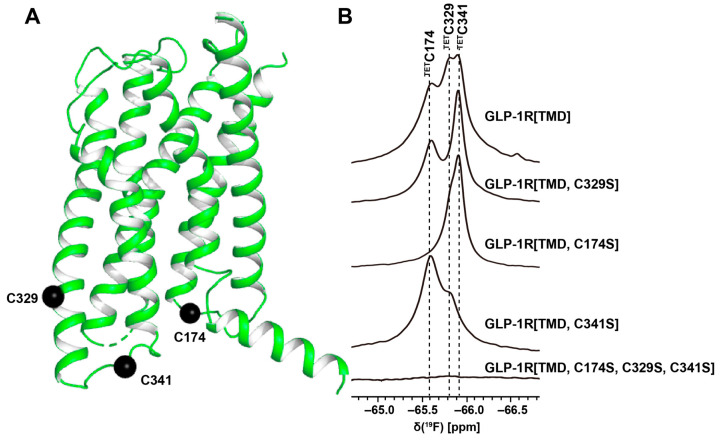
Sequence-specific assignment of the ^19^F-NMR signals of TET moieties attached to the three native Cys residues near the intracellular surface of the transmembrane domain (TMD) of the GLP-1R. (**A**) Crystal structure of the GLP-1R[TMD] (PDB: 5VEX; generated using the PyMOL Molecular Graphics System, Schrödinger, LLC, New York, USA) shown as a green cartoon. Three native Cys residues, C174, C329 and C341, shown as black spheres, are exposed on the intracellular surface and are thus accessible for TET labeling with the IMCM method [56]. (**B**) 1D ^19^F-NMR spectra. The GLP-1R[TMD] spectrum contains three signals corresponding to the three Cys sites shown in (**A**). Individual assignments of the ^19^F-NMR signals were obtained using site-specific mutagenesis to replace cysteines with serine residues. The peak assignments indicated at the top by the one-letter amino acid code and the residue number are based on the disappearance of the signals of the replaced cysteines. (Adapted from Figure 1 in reference [57]).

Fluorine has been widely incorporated into proteins by post-translational chemical modification (Figure 3B). The most commonly employed method is cysteine-labeling, making use of the high nucleophilicity of the side chain sulfhydryl group [43]. Labels with CF_3_ groups are attractive because they yield strong signals that are not subject to large chemical shift anisotropy relaxation [44]. Examples are 3-bromo-1,1,1-trifluoroacetone (BTFA) [52] and 2-bromo-4-(trifluoromethyl)acetanilide (BTFMA), which react with the sulfhydryl group in a single step [53]. The conjugation of 2,2,2-trifluoroethanethiol (TET) to membrane proteins starts with sulfhydryl group activation by 4,4-dithiodipyridine (4-DPS), and a disulfide bond is formed in a second step [42,54,55,56,57]. Post-translational chemical modification can be applied to otherwise unlabeled proteins and regardless of the expression system used; high expression yields can thus be obtained, which is especially useful for GPCRs [24,42,54,55,56,57]. When using amino-acid-specific labeling, further sequence-specific assignments of ^19^F-resonances have been obtained by site-specific mutagenesis. For cysteine-rich GPCRs, individual assignments can therefore be very demanding. In 2015, Sušac et al. [56] reported the in-membrane chemical modification (IMCM) method, which makes use of the natural protection of most cysteines in the transmembrane helices by the membrane environment. Selective cysteine labeling on the receptor surface with minimal or no mutagenesis can thus be achieved. As a recent example, Figure 4 shows sequence-specific ^19^F-NMR assignments based on the IMCM method for a class B GPCR [57]. In this study, three natural cysteines on the intracellular surface of the transmembrane domain of the glucagon-like peptide-1 receptor (GLP-1R[TMD]) were accessible for IMCM; once each of them was individually mutated to serine, sequence-specific assignments were obtained from comparison to the 1D ^19^F-NMR spectra (Figure 4). This provided a basis for studies of conformational changes caused by the bound negative allosteric modulator (NAM) NNC0640 and other ligands [57].

The introduction of multiple ^19^F-labels within the same protein has been used to check on intramolecular distances related to the three-dimensional molecular structure [58,59].

In a genetic engineering approach, the site-specific incorporation of fluorinated amino acids is accomplished through using an orthogonal amber suppressor tRNA with a paired tRNA synthetase to insert the non-proteinogenic amino acid at positions defined by a TAG amber codon (Figure 3C) [40]. Fluorinated phenylalanine [60,61,62] and tyrosine [63] derivatives have been incorporated into proteins using this approach. Genetic labeling can be highly precise, but in improperly optimized expression systems, it may provide low yields of both the expression and incorporation of the fluorinated-amino acid [64]. Wang et al. [62] reported the genetic labeling of the cannabinoid receptor 1 (CB1) with the non-proteinogenic amino acid 3’-trifluoromenthyl-phenylalanine (mtfF) in the baculovirus expression system; this approach enabled studies of conformational transformations under the influence of ligands with variable efficacies [62]. 

## 4. NMR in Solution of GPCRs Using Stable-Isotope Labeling

Three different stable-isotope labeling strategies have primarily been used for studies of GPCRs: post-translational chemical labeling with ^13^C-labeled methyl groups, amino-acid-type selective labeling and uniform labeling. Post-translational chemical labeling of the reactive side chains of surface-accessible cysteine or lysine residues has been used in many NMR studies of GPCRs. ^13^C-isotope-labeled methyl probes can be chemically attached to the γ-SH moiety of cysteine side chains or the ε-NH_2_ groups of lysine side chains. ^13^C-formaldehyde has been used to label solvent-exposed lysines, yielding ^13^C-dimethyllysines as NMR probes [65,66,67]. ^13^C-methyl methanethiosulfonate (^13^C-MMTS) has been used to label solvent-exposed cysteines of GPCRs [68]. Unlike trifluoromethyl probes, which are usually attached to a single judiciously selected surface-accessible cysteine to avoid signal interference from other labeled residues [42,69,70,71,72], ^13^C-labeled methyl probes have often been used to label all surface-accessible (endogenous as well as non-endogenous) cysteines or lysines, and 2D ^1^H-^13^C correlation spectra were recorded to resolve multiple signals [73]. 

Limitations arise because the choice of stable-isotope probes for the chemical modification of amino acids side chains may influence the dynamics of GPCRs [71]. On principal grounds, post-translational chemical labeling normally only targets surface-accessible residues of GPCRs. In contrast, amino-acid-selective isotope labeling also targets the transmembrane region of the receptor. Examples of amino-acid-type selective isotope labeling include the use of [δ_1_-^13^CH_3_]-isoleucine [74,75], [ε-^13^CH_3_]-methionine [28,67,68,74,76,77,78,79,80,81,82,83], [^15^N]-valine [84,85] and [^15^N]-leucine [86]. An increased sensitivity was achieved by deuterating the α- and β- positions of methionine and leucine, using [2,3,3-^2^H, methyl-^13^C]-methionine [28,76,78,79] and [2,3,3-^2^H, ^15^N]-leucine [86] in the nutrient.

**Figure 5:**
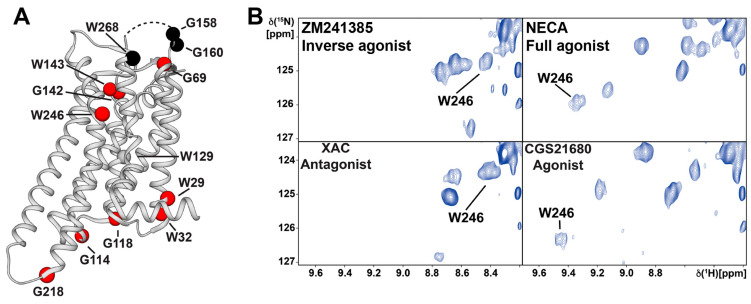
Spectral region containing the Trp246^6.48^ indole ^15^N–^1^H signal of 2D [^15^N, ^1^H]-TROSY correlation spectra of [u-^15^N, ~70% ^2^H]-A_2A_AR in complexes with ligands of different efficacies. (**A**) Locations of glycines and tryptophans in the crystal structure of A_2A_AR in complex with the inverse agonist ZM241385 (PDB: 3PWH), for which NMR signals were assigned. Assigned residues which show a response in their NMR signals to bound ligands with different efficacies are depicted by red spheres. Residues that showed no response are depicted by black spheres. The disordered portion of the ECL2 that was not observed in the crystal structure is shown as a dashed line. (**B**) The indole ^15^N–^1^H NMR signal of the toggle switch tryptophan W246^6.48^ is highly responsive to variable drug efficacy, as observed by comparing the spectra for A_2A_AR in complexes with the inverse agonist ZM241385, the antagonist XAC, the selective agonist CGS21680 and the full agonist NECA. (Adapted from Figure 4D in reference [88]).

Uniform ^15^N-labeling of GPCRs has been achieved in *E. coli* for the rat neurotensin receptor 1 [87] and in *Pichia pastoris* for A_2A_AR [88,89,90] and the histamine H_1_ receptor [91]; the minimal medium contained [^15^N]-ammonium sulfate or [^15^N]-ammonium chloride as the only nitrogen sources. Since deuteration is mandatory for transverse relaxation-optimized spectroscopy (TROSY) studies of large macromolecular systems [92], it is essential that the expression systems used can produce partially or fully deuterated recombinant proteins. Uniform ^2^H, ^15^N-labeling was also achieved by expression of the β_1_-adrenergic receptor in *Sf9* insect cells; the addition of ^2^H, ^15^N-labeled yeast extract to the insect cell medium allowed deuteration levels of >60% [93]. Eddy et al. [88,89,90] expressed uniformly ^2^H, ^15^N-labeled A_2A_AR with D_2_O-adapted *Pichia pastoris* in D_2_O growth media. All six tryptophan indole ^15^N-^1^H signals and eight of the eighteen glycine backbone ^15^N-^1^H NMR signals (Figure 5A) were resolved in the 2D [^15^N, ^1^H]-TROSY spectrum of A_2A_AR, and sequence-specific NMR assignments were obtained by single-residue amino acid replacements [88]. Drug-dependent local conformational changes in A_2A_AR could thus be observed (Figure 5B), as illustrated for the toggle switch Trp246^6.48^ in Figure 5 [88]. In addition to the natural tryptophans, extrinsic tryptophan residues were introduced into judiciously selected sites of the receptor by genetic engineering; these were then used as supplementary NMR probes for monitoring conformational changes of A_2A_AR [89,90]. 

Overall, in contrast to “probe methods”, uniform labeling can provide global information on a receptor; in Figure 5A, this is visualized by the distribution of the residues observed in studies of A_2A_AR [88]. Uniform stable-isotopic labeling has also been used for a de novo structure determination of the seven-helix transmembrane receptor rhodopsin II from *Natronomonas pharaonic* [94], but no de novo structure determination has as yet been reported for a human GPCR.

## 5. GPCR–Ligand Interactions Studied by NMR Observation of the Ligand

NMR observation of bound and free ligands can provide unique insights into the biophysical properties and biological functions of GPCRs. Specifically, in addition to providing data on the influence of bound ligands on GPCRs, as described in the above Section 3 and Section 4, NMR spectroscopy is uniquely powerful in detecting weak binding [95,96,97]. Measurements of the chemical shifts, line widths, and relaxation times of free and bound ligands are all informative on ligand binding events. Depending on the time scale of the GPCR–ligand interactions, different approaches are used [98]. 

**Figure 6:**
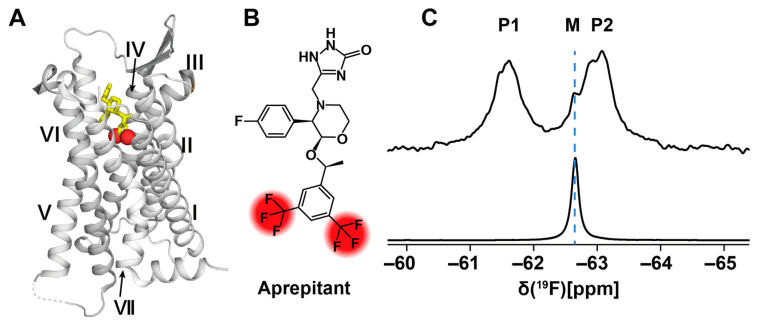
^19^F-NMR observation of the ligand aprepitant bound to NK1R. (**A**) Crystal structure of the NK1R complex with aprepitant (PDB: 6J20). NK1R is shown in ribbon presentation with grey color. Aprepitant is shown in yellow stick presentation, with red spheres representing the trifluoromethyl groups. The transmembrane helices are identified with roman numerals. (**B**) Chemical structure of the drug aprepitant. The two –CF_3_ groups are highlighted in red. (**C**) 1D ^19^F-NMR spectra of aprepitant in complex with NK1R (upper trace) and “free” in DDM/CHS micelles (lower trace). M is the ^19^F-NMR signal of micelle-associated “free” aprepitant; P1 and P2 are the two –CF_3_ signals of NK1R-bound aprepitant. (Adapted from Figure 4, A and E, in reference [99]).

Figure 6 illustrates an NMR study of a stably bound ligand which is in slow exchange. In the free state in isotropic surroundings, the ligand aprepitant shows a single NMR signal of the two –CF_3_ groups (Figure 6B). The impact of the neurokinin 1 receptor (NK1R) on the bound aprepitant is to separate the resonances of the trifluoromethyl groups into two peaks (Figure 6C). The chemical shift difference between the resulting signals P1 and P2 is due to different micro-susceptibilities at the positions of the two trifluoromethyl groups (Figure 6B) in the NK1R orthosteric ligand binding groove. From ring current calculations based on the crystal structure of the NK1R–aprepitant complex, the two signals were individually assigned [99]. The data in Figure 6 then enabled studies of the dynamics of the NK1R–aprepitant complex, which revealed that the orthosteric binding groove undergoes transient fluctuations with an outstandingly large amplitude [100]. 

For studies of weak binding with a rapid ligand exchange, a large arsenal of experiments based on observation of the modulation of the NMR signal of the free ligand through exchange with the bound ligand is available [101,102,103,104]. Among these, the transfer NOE (trNOE) stands out by the fact that information on the structure of the bound ligand can be obtained. For example, in studies of the peptide ligand dynorphin interacting with the kappa opioid receptor (KOR) [105], ^1^H and ^15^N chemical shift variations for the free ligand indicated that the free peptide is in fast exchange with the bound peptide. The receptor–peptide interaction was within the range that allowed the determination of a conformation of the KOR-bound dynorphin via the trNOE method.

## 6. Conclusions and Outlook

This brief overview recalls that with the use of dedicated labeling methods, NMR spectroscopy has an attractive role in the structural biology of GPCRs bound to drug ligands with different efficacies, in spite of the inherent complexity of these systems. 

NMR measurements in solution complement the molecular architectures obtained with X-ray crystallography and cryo-EM by providing information on GPCR structural dynamics, ligand interactions with GPCRs and ligand-induced conformational changes in GPCRs [106]. Although a de novo structure determination of a GPCR by NMR has been described [94], future NMR studies can be expected to focus primarily on structural dynamics and ligand interactions. Progress will be largely dependent on the development of further methods for the site-specific introduction of ^19^F-NMR probes and stable-isotope labels. 

In spite of the amazing results from cryo-EM studies during the last few years [107,108,109,110,111,112,113,114], obtaining atomic resolution information on GPCR–ligand interactions and on function-related transient structure fluctuations in solution at ambient temperatures remains unique to NMR. NMR spectroscopy will therefore continue to have an important role in the arsenal of methods used in the study of the GPCR’s structural biology.
